# Supplementary material for: Powder-size driven facile microstructure control in powder-fusion metal additive manufacturing processes
Source: Nat Commun. 2024 Apr 11;15:3094. doi: 10.1038/s41467-024-47257-w (PMC11009264; doi:10.1038/s41467-024-47257-w)
Supplement: Supplementary file 1 — Supplementary Information [file 41467_2024_47257_MOESM1_ESM.pdf]

Supplementary information for

Powder-size driven facile microstructural control in powder-fusion metal AM processes

Shubham Chandra[1], Chengcheng Wang[1], Shu Beng Tor[2], Upadrasta Ramamurty[2,3], Xipeng Tan[4,5]*

[1] Singapore Centre for 3D Printing, School of Mechanical and Aerospace Engineering, Nanyang Technological University, 50 Nanyang Avenue, Singapore 639798
[2] School of Mechanical and Aerospace Engineering, Nanyang Technological University, 50 Nanyang Avenue, Singapore 639798
[3] Institute of Materials Research and Engineering, Agency for Science, Technology and Research, 138634, Singapore
[4] Department of Mechanical Engineering, National University of Singapore, 9 Engineering Drive 1, Singapore 117575
[5] Department of Materials Science and Engineering, National University of Singapore, 9 Engineering Drive 1, Singapore 117575

*Corresponding author. E-mail: xptan@nus.edu.sg

Supplementary Notes

1. Inventive engineering of FG equiaxed microstructure using E-PBF process

Though the impact of PSD on the 3D-printed microstructure is evident from our DED fabricated 316L samples from fine-, coarse-, and FC-powder feedstock thus providing us with reigns for in-situ microstructure control using particle size-driven melt pool engineering (MPE) approach, achieving the extreme CET in bulk samples as seen in our E-PBF samples required a bit of ingenuity by simultaneously exploiting the improvement in the powder-bed thermophysical properties due to the coarse PSD and the algorithm in the ARCAM's EBM® system that is programmed to maintain a predestined build temperature throughout the printing process. First, we determined the optimum process parameters needed to fabricate completely dense 316L SS parts from the EBM® system using fine powders. Then we utilized coarse powders as feedstock while engaging the ARCAM's automated mode algorithm – 1D analyze, for the process parameters optimized with fine powders and a build temperature of 850 °C. Due to the superior thermophysical properties of the coarse powder bed over the fine-powder one (determined by laser flash apparatus (LFA) and differential scanning calorimetry (DSC), as shown in Supplementary Figure 3a), heat transfer in the initial stages of 4 – 5 layers of part fabrication was much higher than fine powder as evident from the thermocouple measurements recorded for the two powders (see Supplementary Figure 4a). Hence, when using the coarse powders, we in fact "tricked" the algorithm into believing it was printing a different material with much higher thermal diffusivity, hence, it slowed down the beam scanning speed substantially from ~ 6 – 10 m s^{-1} in case of fine powders to ~ 2 – 4 m s^{-1} to maintain the designated temperature (Supplementary Figure 4b). This too when the thermophysical properties of the deposited 316L part remained independent of the feedstock utilized (Supplementary Figure 3b). A lower beam scanning speed for the deposited SS316L implied a far lower thermal gradient during solidification – as evident from ~ 1.5 times deeper melt pool and the numerical simulation results subjected to the same cooling rate – given the constant

power and same build temperature and evident from the same PDAS observed in fine and coarse powder 316L SS samples. Hence the solidification rate increased substantially for coarse powder samples resulting in the CET during melt pool solidification.

2. Fine- and coarse-powder single tracks were deposited using the L-DED process.

The single tracks were deposited using fine and coarse powders through the BeAM Magic 800 L-DED process using the parameters provided in Supplementary Table 1. The grain size and aspect ratio parameters obtained from the EBSD maps were summarized in Fig. 2a with the complete dataset provided in Supplementary Figure 5, Supplementary Figure 6, and Supplementary Figure 7 in the order of increasing power, scanning speed, and powder flow rate, respectively. The parameter sets are denoted with the #-symbol. From Supplementary Figure 5 we can notice the effect laser beam power has on the melt pool architecture, bead shape, and microstructural evolution. We can notice a drastic increase in the bead deposition efficiency for both coarse and fine powders with increasing power. However, coarse-powder beads show deeper melt pools specifically for #4 and #5. As seen in Supplementary Figure 6, the melt pool architecture for coarse and fine powders shows minimum differences for a range of scanning speeds. This is also evident from the dimension chart shown in Fig. 2D of the main text.

3. EBSD maps obtained for fine- and coarse-powder feedstocks.

Grain sizes of 2.3 ± 0.2 µm and 6.2 ± 0.4 µm were measured from the EBSD mapping of the fine- and coarse-powder, respectively – shown in Supplementary Figure 8. It is interesting to note that the fine powders also have significantly finer grains in the feedstock compared to the coarse powders.

4. Impact of particle size and time of flight on its temperature rise in DED

We employed a heat balance equation detailed in earlier research [1] on DED processing. The equation could be implemented in our application with the following assumptions. One, the laser intensity at its focal plane is assumed to follow the top-hat profile, which is true for a Nd:YAG fibre laser – the same as the one employed by us for the fabrication of DED samples. Another assumption is that there is negligible thermal gradient within the powder particle and there is no shielding effect by particles lying in a higher vertical plane. We also must assume that only the upper hemispherical surface of the spherical powder is heated and the laser is an uninterrupted energy source. With these assumptions in place, we can write the energy balance equation [1] as:

$$\left(\frac{4}{3}\pi r_p^3\right).\rho_p C_p^p \Delta T = \alpha_p \left(\frac{P}{\pi r_b^2}\right).\left(2\pi r_p^2\right).t_f \tag{2}$$

$$or, \qquad \Delta T = \left(\frac{3\alpha_p P}{2\pi r_b^2 \rho_p C_p^p}\right).\frac{t_f}{r_p} \tag{3}$$

where r_p is particle radius, ρ_p is particle density, C_p is the specific heat capacity of the powder material, ΔT is the temperature rise, α_p is particle absorptivity, P is power, r_b is the spot radius, and t_f is the time of flight.

For a PSD containing N number of particles with masses m_1, m_2, $m_3 \ldots m_N$ and their respective mass fractions in the PSD given as x_1, x_2, $x_3 \ldots x_N$ where the respective preheat temperatures have been determined using Supplementary Equation (3), rise in energy of a single particle in Joules can be given as:

$$E_i = m_i C_p \Delta T_i \tag{4}$$

The total energy rise of the N particles in the PSD can be given by the following equation:

$$E_{total} = \sum_{i=1}^{N} m_i x_i C_p \Delta T_i \tag{5}$$

The total energy rise/unit mass of the PSD in Joule kg^{-1} can then be calculated as:

$$E = \frac{\sum_{i=1}^{N} m_i x_i C_p \Delta T_i}{\sum_{i=1}^{N} m_i x_i} \tag{6}$$

As the heated feedstock is incident to the melt pool with a certain mass flow rate, m_f (kg s^{-1}), the energy incident to the melt pool per second will be given as:

$$\dot{E} = E \times m_f \tag{7}$$

The $\dot{E}$ value obtained from Supplementary Equation (7) is the equivalent power of a secondary heating source to the melt pool which can be determined for a feedstock PSD and later be employed for numerical simulations. The $\dot{E}$ values for the fine and coarse powder feedstocks employed by us for a powder flow rate of 3.25 g min^{-1} were obtained as 85.2 W and 92.0 W, respectively.

5. Machine learning model framework

Supplementary Figure 9 shows the temperature (Supplementary Figure 9a) and heat flux (Supplementary Figure 9b) contour plots for a PSD obtained through the Mote3D-Abaqus® coupling in this work. The heat flux contour – HFL.HFL2 provided the flux values directed along the y-direction in the simulation domain directed from top to bottom. The average thermal conductivity from the simulated powder bed was determined by averaging the heat flux through the domain and using Fourier's law of heat conduction as below:

$$q = -k \nabla T \tag{8}$$

where q is the heat flux in W m^{-2}, k is the thermal conductivity in W $m^{-1}K^{-1}$, and ∇T is the thermal gradient across the domain in K m^{-1}.

The powder bed density and thermal conductivity values measured for 20 PSDs employed to develop the ML framework using the Mote3D-Abaqus® coupling are provided in Supplementary Table 2. The datasets employed for testing the ML model along with their predicted values can be seen in Supplementary Table 3.

Two identical multilayer perceptron (MLP) models were built to train and predict the mean and deviation values for powder size distribution separately. The inputs were thermal

conductivity and packing density. The ML model herein consists of two hidden layers, each with 50 neurons and activated by a ReLU function. A dropout rate of 0.2 was implemented as a regularization technique following the hidden layer to reduce overfitting caused by the small data set. Linear activation function was used in the output layer. An adaptive stochastic gradient descent optimizer, namely Adam [2] with a learning rate of 1×10^{-3} and decay of $0.002 \times e^{-3}$ was employed to update the MLP training weights (i.e. the coefficients of connectivity between neurons in adjacent layers) amid backpropagation. Mean squared error was selected as the loss function. The model was trained using 500 epochs with a mini-batch size of 4. The train/test split ratio was 80/20, as a common practice in ML. All the above hyperparameters were tuned empirically. Batch size is an important hyperparameter. Using mini batches achieves the best training stability and avoids high computational costs as well as local minima. During the training, checkpoints were called to automatically save the model weights with the lowest validation loss. The result shows an average root mean squared error of 9.6 and 7.5 for mean and deviation prediction of the test data, respectively.

6. DPM simulations of powder flow through the nozzle in L-DED.

Supplementary Figure 10 shows the modelled nozzle geometry and the particle flow results obtained from the DPM simulations. The nozzle assembly in the BeAM Magic 800 system is comprised of three nozzle cones. The powder flow with the carrier gas takes place through the gap between the outermost and the intermediate nozzle (labelled as outer and inner nozzles, respectively) modelled in Supplementary Figure 10a. A sectional view of this assembly presented in Supplementary Figure 10b shows the region of interest (or simulation domain) labelled as 'powder flow region'. The particle trajectory plots presented in Supplementary Figure 10c have particles with colour coding that corresponds to their velocity magnitudes provided in the legend. The outcome of these simulations charting the variations in the vertical flow velocities and time of flight with particle sizes has been provided in Supplementary Figure 14. The impact of this difference on the resulting melt pool solidification parameters is evident from the 2D heat transfer simulations accounting for the heat flux incident to the melt pool from the fast (or not so fast) moving powder particles.

7. Macro-scale solidification simulations of the L-DED and E-PBF process.

Numerical simulations to model the melt pool solidification in a single-track for L-DED and E-PBF deposited SS316L were performed using the COMSOL v5.2 finite element analysis simulation package. The melt pool melting and solidification were simulated in a 2D domain with the implementation of analytical models for the heat source. The heat flux from the incident beam was implemented as follows in the form of a boundary heat source:

$$q = \frac{2 \cdot \alpha \cdot P}{\pi \cdot r_0^2} exp\left(-2\left(\frac{t - t_0}{t_0}\right)^2\right) exp\left(-2\left(\frac{x - x_0}{r_0}\right)^2\right) \quad (9)$$

where α is the beam absorptivity, r_0 is the spot size radius of the beam, P is the operating power, t_0 and x_0 are the time instance and the position where the peak of the Gaussian energy distribution is meant to peak. For the 2D domain chosen for the simulations, the movement of the heat source is taken to be in the plane perpendicular to the domain. Hence, the pseudo-3D

distribution of the heat flux requires both the time and the dimension factor as in the exponential function above in Supplementary Equation (9).

For simulation of the E-PBF process, a region 70 μm thick representing the sintered powder-bed region is chosen over the prebuilt domain with diminished thermophysical properties. Specific to the E-PBF process, the sintered powder bed and the domain representing the deposited material were preheated to 850 deg. C and the process parameters obtained in Supplementary Figure 4b. The thermal history and gradient were recorded at probes in the middle of the melt pools with the movement of the heat source.

For the fine and coarse powder E-PBF process simulations, a time step of 0.1 μs was employed for a total simulation time of 5 ms. An initial mesh was generated using predefined metrics of 'Fine' and calibrated for fluid dynamics in the COMSOL Multiphysics software. For this setting, the element size range was from 5 – 175 μm. Further, the region below the top surface – where the simulated heat flux was incident, had a set of 40 boundary layers having a stretching factor of 1.1 with the smallest boundary layer thickness of 3 μm just below the surface. Adaptive mesh refinement was enabled with the time-dependent linear (PARDISO) solver employed for the heat transfer simulations. The smallest mesh size of 1.5 μm was obtained at the end for coarse powder simulations while 2.8 μm was obtained at the end for fine powder simulations.

For the simulation of the L-DED process, an added heat flux due to the powder particles was employed considering the preheated powders as a secondary heat source as follows:

$$q_p = \frac{2 \cdot \eta_c \cdot \dot{E}}{\pi \cdot r_0^2} exp\left(-2\left(\frac{t - t_0}{t_0}\right)^2\right) exp\left(-2\left(\frac{x - x_0}{r_0}\right)^2\right) \tag{10}$$

where, $\dot{E}$ is determined by the Supplementary Equation (7), η_c is the powder catchment efficiency determined by the mass of the deposited bead (density × cross-sectional area × length) divided by the mass of the powder incident to the melt pool during the time taken for the deposition. The $\dot{E}$ values for the fine and coarse powder feedstocks employed by us for a powder flow rate of 3.25 g min^{-1} were obtained as 85.2 W and 92.0 W.

A time step of 1 μs was employed for a total simulation time of 50 ms for both the coarse and fine powder simulations for the L-DED process. The initial meshing parameters were the same as that for the E-PBF simulations except that the adaptive meshing resulted in the smallest element size of 10.8 μm for both fine and coarse powder simulations. The thermal history and thermal gradient values were determined at a point in the middle of the melt pool – 200 μm below the free surface.

The solidification parameters obtained for the E-PBF and L-DED simulations are shown in Supplementary Figure 11.

8. <u>Solidification maps for the L-DED and E-PBF processes.</u>

As the primary dendrite arm spacing (PDAS) was measured as 1.25 μm, for both fine and coarse powder samples obtained from E-PBF (Supplementary Figure 12b and d), the cooling

rate during the melt pool solidification can be calculated for SS316L by the following equation [3]:

$$\lambda_1 = 80 \times (cooling\ rate)^{-0.33} \quad (11)$$

where λ_1 is the PDAS in μm and the *cooling rate* value is in K s^{-1}. From this equation, the *cooling rate* was determined as 3.0×10^5 K s^{-1}. The thermal gradient magnitude values were determined from the macro-scale numerical simulations as 4.7×10^6 K m^{-1} and 8.5×10^5 K m^{-1} for fine and coarse powder samples, respectively, at the advent of solidification. As the point at which the G and *cooling rate* values were obtained lies in the midway of the melt pool width, the velocity of the solidification front, or growth rate (*V*), at this point is directed along the build direction. Its value can be determined by the relation: $V = Cooling\ rate/Thermal\ Gradient$. Thus, for the fine and coarse powder samples, V is obtained as 0.064 m s^{-1} and 0.353 m s^{-1}, respectively.

L-DED depicts a solidification history that is significantly different from that of the E-PBF deposition process. First, the AR value of 2.5 obtained in L-DED fine powder samples, which is considered a borderline for columnar grain microstructure, shows that the melt pool solidification parameters obtained from L-DED process parameters explored by us encourage the formation of equiaxed grains. However, these solidification parameters are influenced by the particle sizes which causes the formation of finer equiaxed grains in coarse powder samples. We observed that a smaller average PDAS value of 0.90 μm is obtained in these samples (Supplementary Figure 12c), indicating that they cooled much faster than the fine powder samples which exhibit a PDAS value of 1.82 μm (Supplementary Figure 12d). Using this information with Supplementary Equation (11) gives us the values of cooling rates for fine and coarse powders as 9.6×10^4 and 7.94×10^5 K s^{-1}, respectively. Hence the melt pool in coarse-powder DED samples solidified at a rate nearly 10 times faster than the fine-powder samples. Employing the time of flight, particle size and particle preheating physics, 2D heat transfer simulations (detailed in Supplementary information) provide a result similar in trend as seen in E-PBF 316L i.e., lower thermal gradient for the coarse powder feedstock. A higher cooling rate coupled with a lower thermal gradient obtained in the DED coarse-powder samples results in a significantly higher melt pool solidification rate causing the CET.

The determination of nucleation density values for the CET curve shown in Fig. 3G of the main text was done based on the equation widely applied for generating process microstructure maps[4,5] where thermal gradient, *G* and solidification rate, *V* remain in the order of 10^6 K m^{-1} and cm s^{-1}, respectively. The equation is written as:

$$\frac{G^n}{V} = a\left(\sqrt[3]{\frac{-4\pi N_o}{3\,ln(1-\varphi)}}\cdot\frac{1}{n+1}\right)^n \quad (12)$$

where N_o is the nucleation density, φ is Hunt's parameter, and *a* and *n* are material constants. The values of these material constants were determined by power law curve fitting of the solidification velocity – undercooling variation curve for SS316L provided in an early work by Lin et al. [6] which were determined through thermodynamic modelling of the rapid solidification

phenomena. The parameters 'n' and 'a' were determined respectively as 2.94 and 5.87E04 $K^{2.94}$ s m^{-1} and were employed for plotting the CET curves. We determined four values of N_o – each for the fine-powder E-PBF, coarse-powder E-PBF, fine-powder L-DED, and coarse-powder L-DED. The thermal gradient value in Supplementary Equation (12) was inserted from the numerical simulation results for the aforementioned 4 cases whereas the solidification rate values were determined from the derived values of cooling rates from the experimentally observed primary dendrite arm spacings, Supplementary Equation (11), and thermal gradient values from the numerical simulations. The Hunt's parameter, φ, or the volume fraction of equiaxed grains was determined experimentally from the EBSD mapping as the percentage of grains with an aspect ratio (length-to-width ratio) ≤ 2.5. Supplementary Table 4 provides the details of the values measured and calculated N_o. The limiting equations that determine the G vs V relation for equiaxed and columnar grain growth, presented in Fig. 3 b of the main text are given as [7]:

$$G \geq G_{col} = 2.9\, N_o^{\frac{1}{3}} \left(1 - \frac{\Delta T_n^3}{\Delta T_c^3}\right) \Delta T_c \qquad (13)$$

$$G \leq G_{eq} = 0.617\, N_o^{\frac{1}{3}} \left(1 - \frac{\Delta T_n^3}{\Delta T_c^3}\right) \Delta T_c \qquad (14)$$

where, ΔT_n and ΔT_c are nucleation and constitutional tip undercoolings, respectively. Constitutional undercooling can be related to the growth rate, V as [4]:

$$\Delta T_c = (a \cdot R)^{\frac{1}{n}}. \qquad (15)$$

The value of ΔT_n was taken as 5 K [8] given the primarily austenitic microstructure obtained in various fine and coarse E-PBF and L-DED samples.

Supplementary Figures

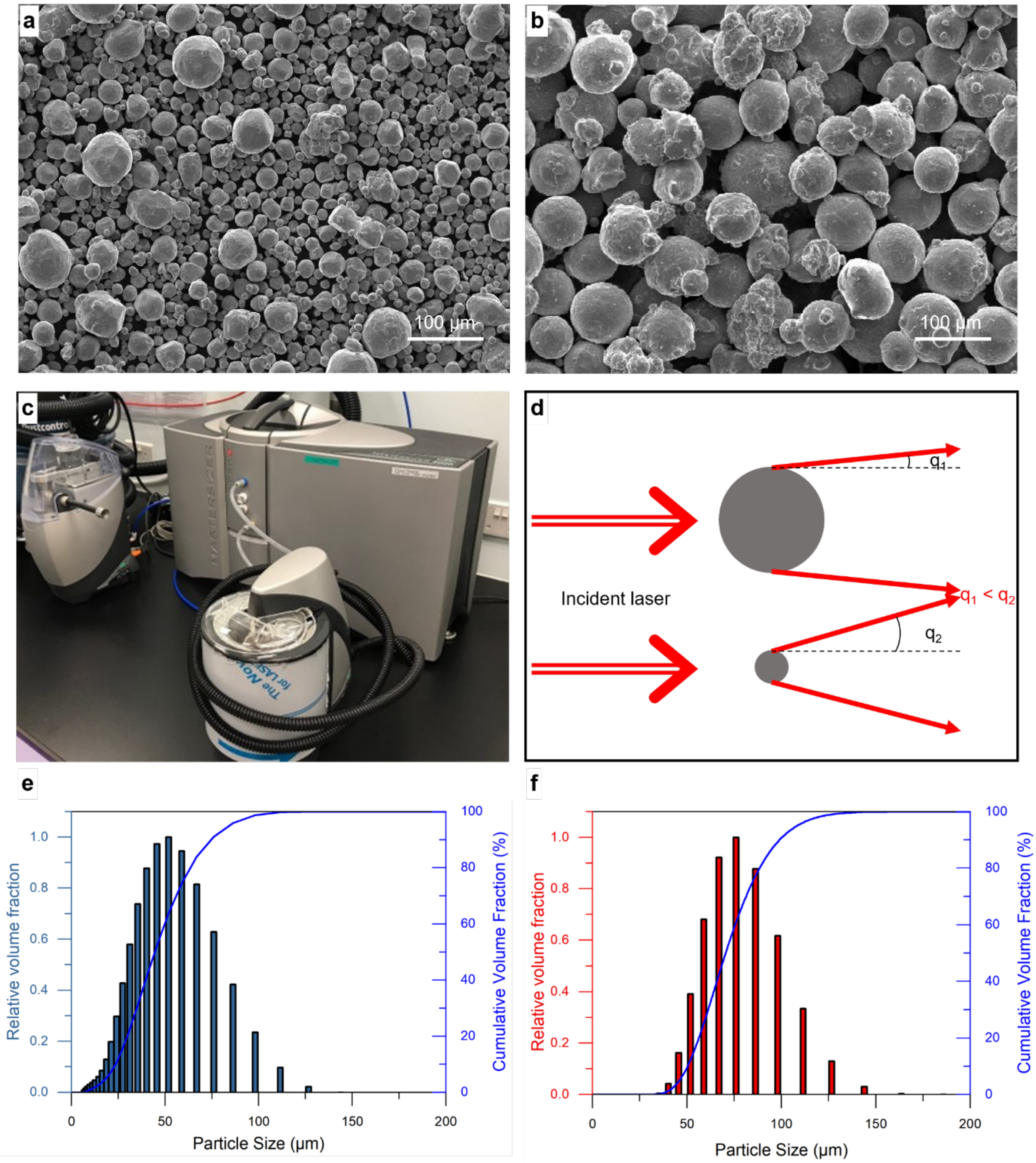

Supplementary Figure 1: Powder feedstocks employed for L-DED and E-PBF processes. Scanning electron micrographs obtained for (a) fine and (b) coarse SS316L powders (c) Malvern Panalytical Mastersizer 3000 with Aero S adapter (d) Principle of Mie scattering employed by the mastersizer for particle size determination. Powder-size distribution obtained from the Mastersizer with cumulative volume fraction for (e) fine and (f) coarse powders.

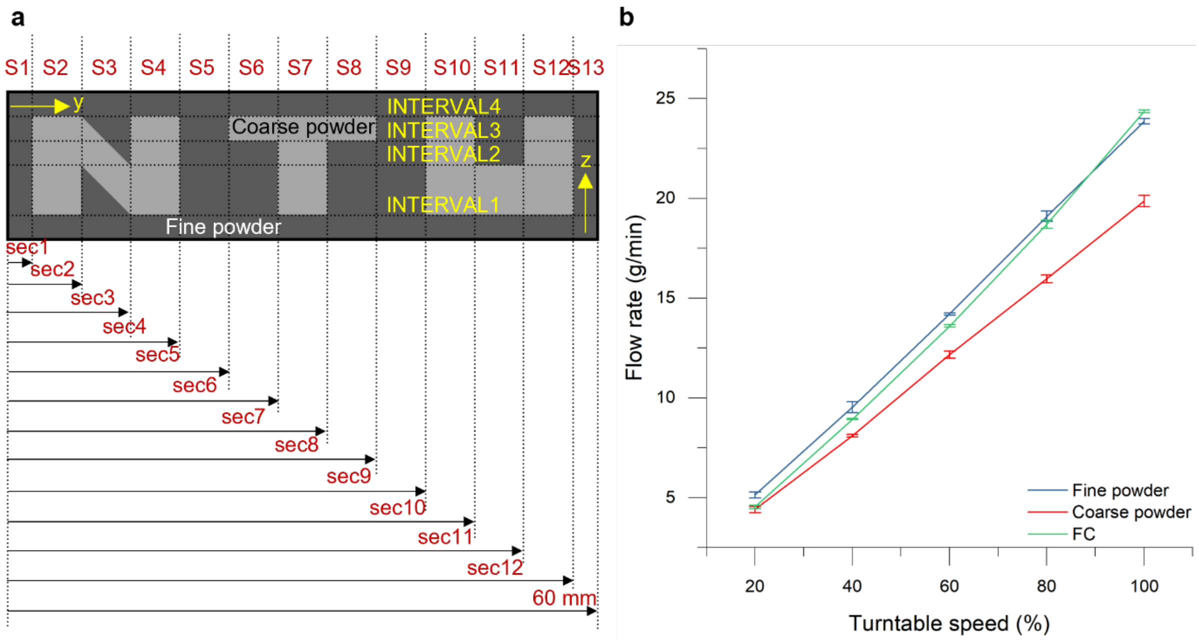

Supplementary Figure 2: DED fabrication with fine-, coarse-, and FC-powder feedstocks. (a) POC part schematic utilized to highlight the in-layer microstructure control achieved through particle size-driven MPE in L-DED. Two hoppers were used simultaneously with fine and coarse granular feedstock and were invoked at different intervals and sections to result in regions with distinct features – divided among 'letter zone' composed of letter N, T, and U printed using coarse powders and 'matrix zone' printed using fine powders. (b) Powder flow rate vs turntable speed curve obtained for fine-, coarse-, and FC-powder feedstocks. The observations of powder flow rate for each of the turntable speeds were repeated at least three times to establish statistical validity of the measurements.

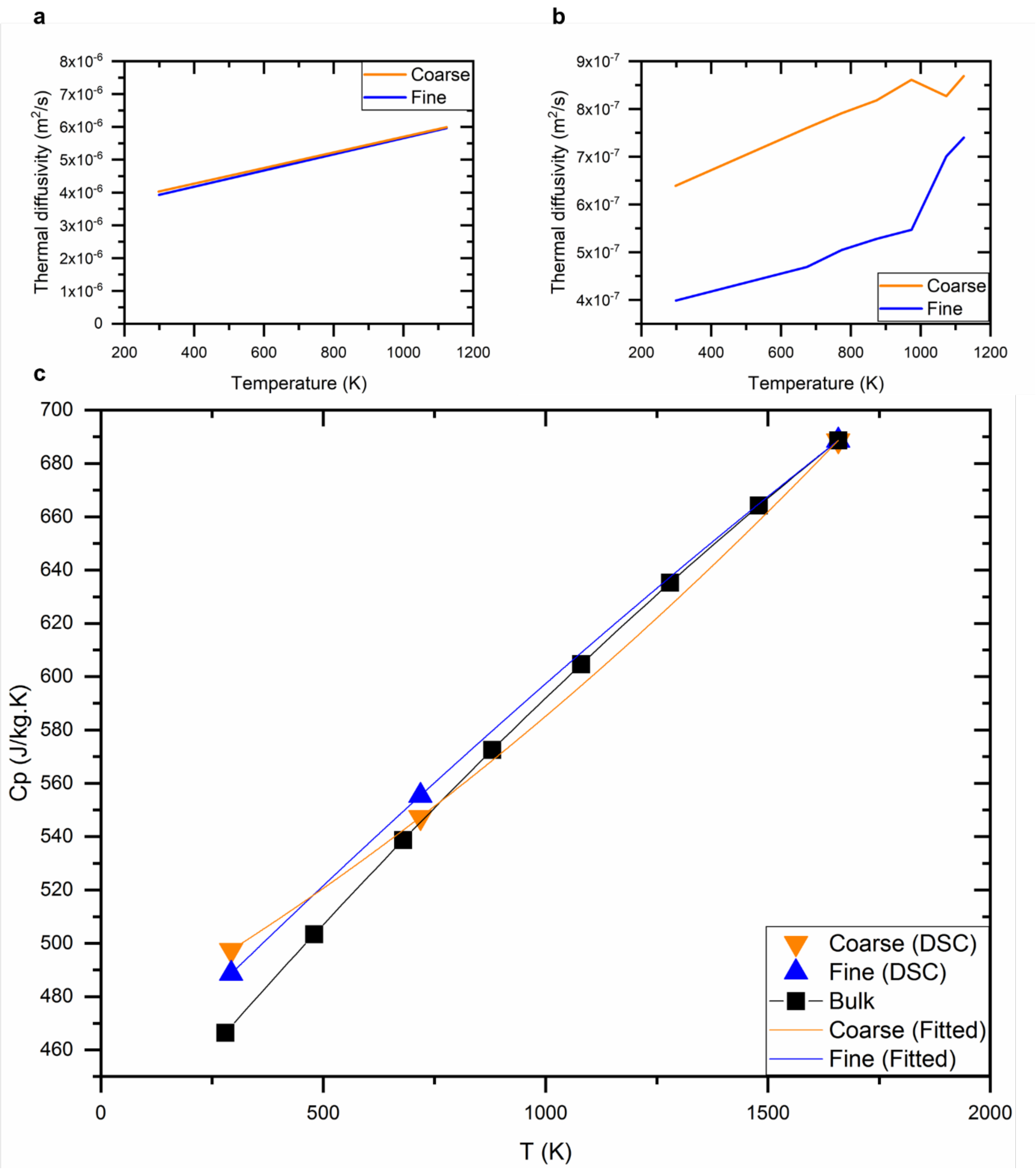

Supplementary Figure 3: Temperature-dependent thermophysical properties of the sintered powder bed and EBM-printed SS316L. Thermal diffusivity measurements of (a) 3D printed samples and (b) sintered powder-bed samples. (c) Specific heat capacity measurements of sintered powder-bed samples.

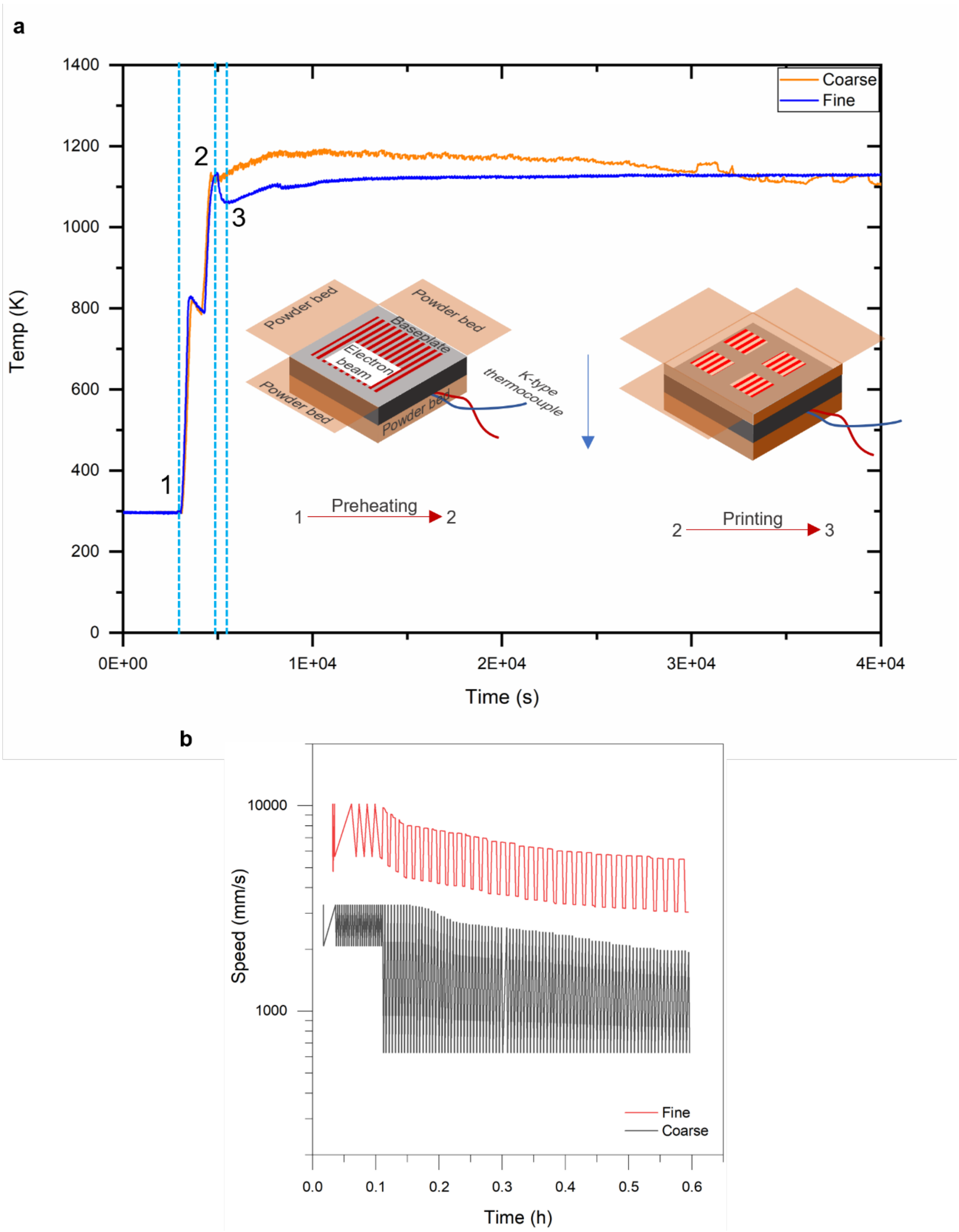

Supplementary Figure 4: Powder size effect during the part fabrication in the ARCAM EBM® system. (a) The data recorded during the printing of fine powders (dark blue curve) shows the typical dip in the recorded temperature after the preheating stage. Coarse powder (orange curve) shows the contradictory behavior that impacts the 1D analyze program to significantly alter the beam scanning speed for the coarse powder bed as shown in (b).

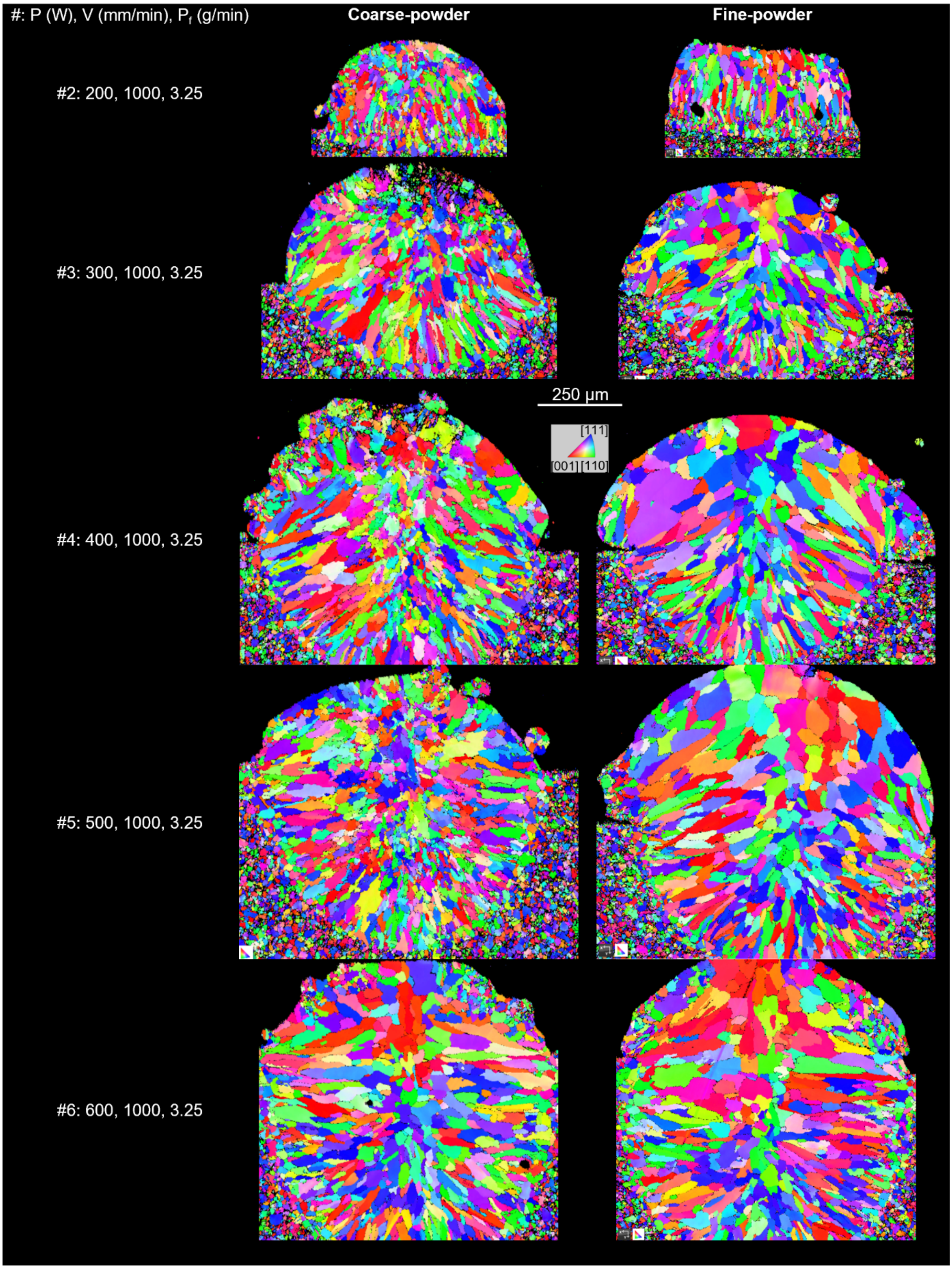

Supplementary Figure 5: Effect of increasing power on the coarse- and fine-powder single-tracks deposited using the L-DED system. The IPFz maps obtained in the transverse section of the single tracks printed using parameters #2 – 6 shown in Supplementary Table 1

with scanning speed and powder flow rate of 1000 mm/min and 3.25 g/min, respectively, and increasing laser power from 200 – 600 W in 100 W intervals.

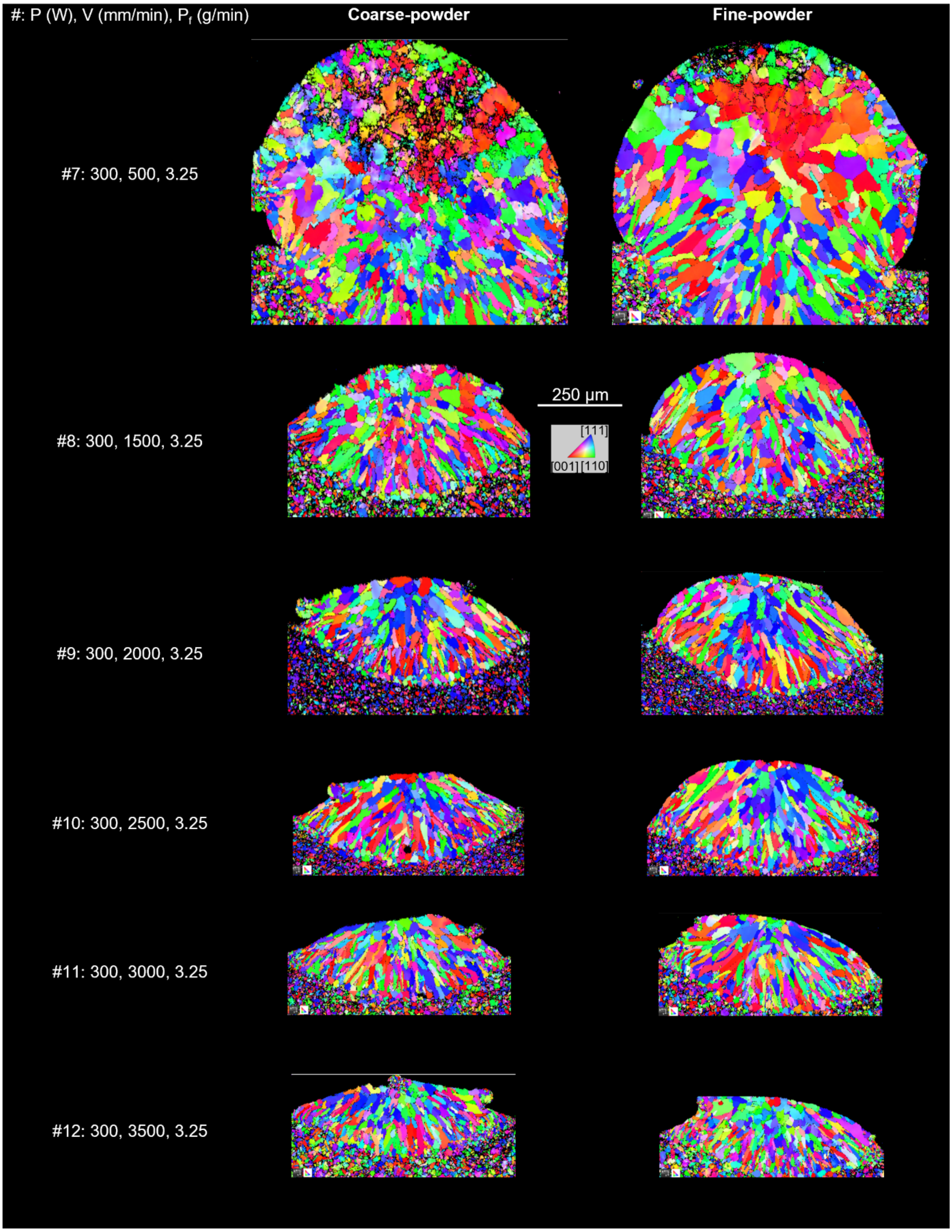

Supplementary Figure 6: Effect of increasing scanning speed on the coarse- and fine-powder single-tracks deposited using the L-DED system. The IPFz maps obtained in the transverse section of the single tracks printed using parameters #7 – 12 shown in Supplementary Table 1 with laser power and powder flow rate of 300 W and 3.25 g/min, respectively, and increasing laser scanning speed.

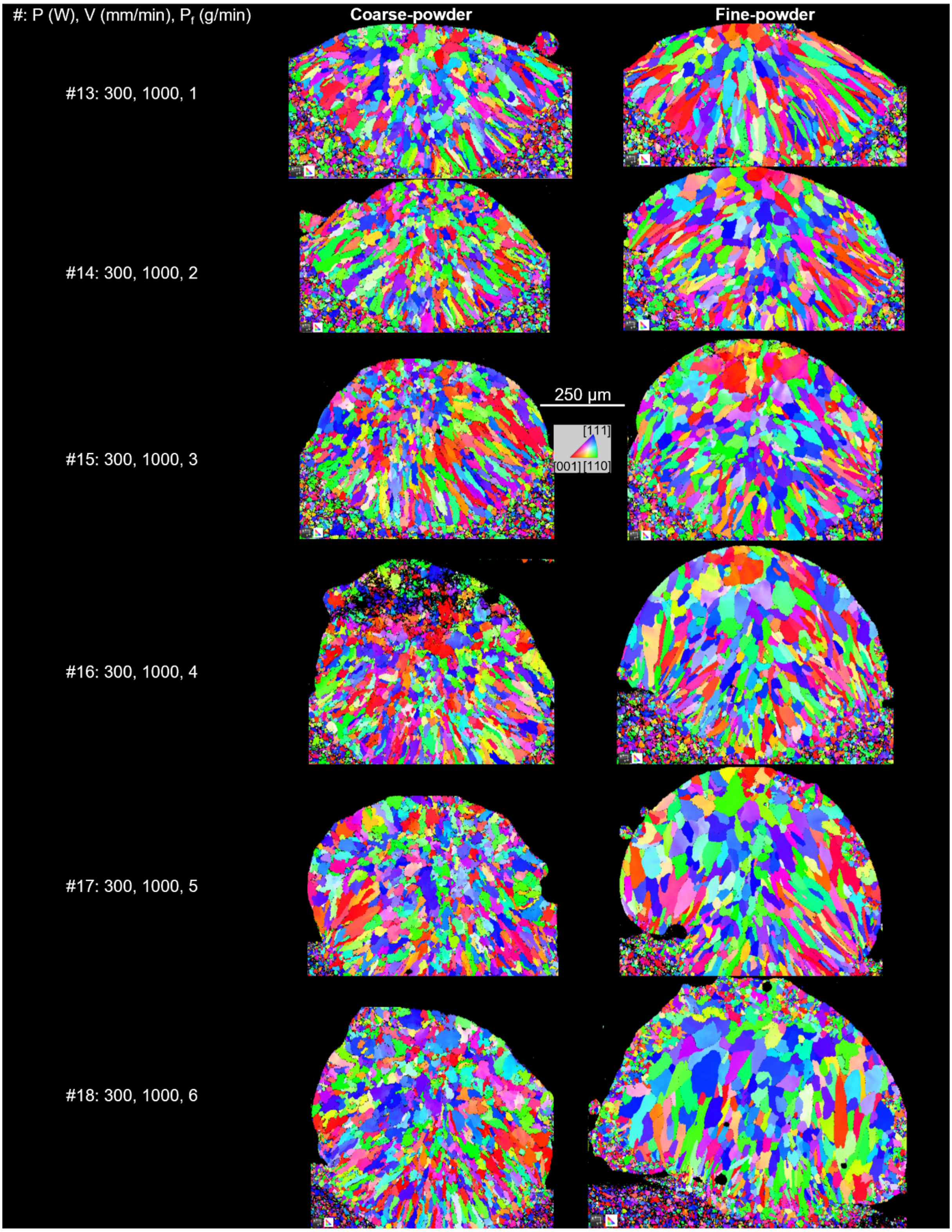

Supplementary Figure 7: Effect of increasing powder flow rate on the coarse- and fine-powder single-tracks deposited using the L-DED system. The IPFz maps obtained in the transverse section of the single tracks printed using parameters #13 – 18 shown in Supplementary Table 1 with laser power and scanning speed of 300 W and 1000 mm/min, respectively, and increasing powder flow rate from 1 – 6 g/min in the intervals of 1 g/min.

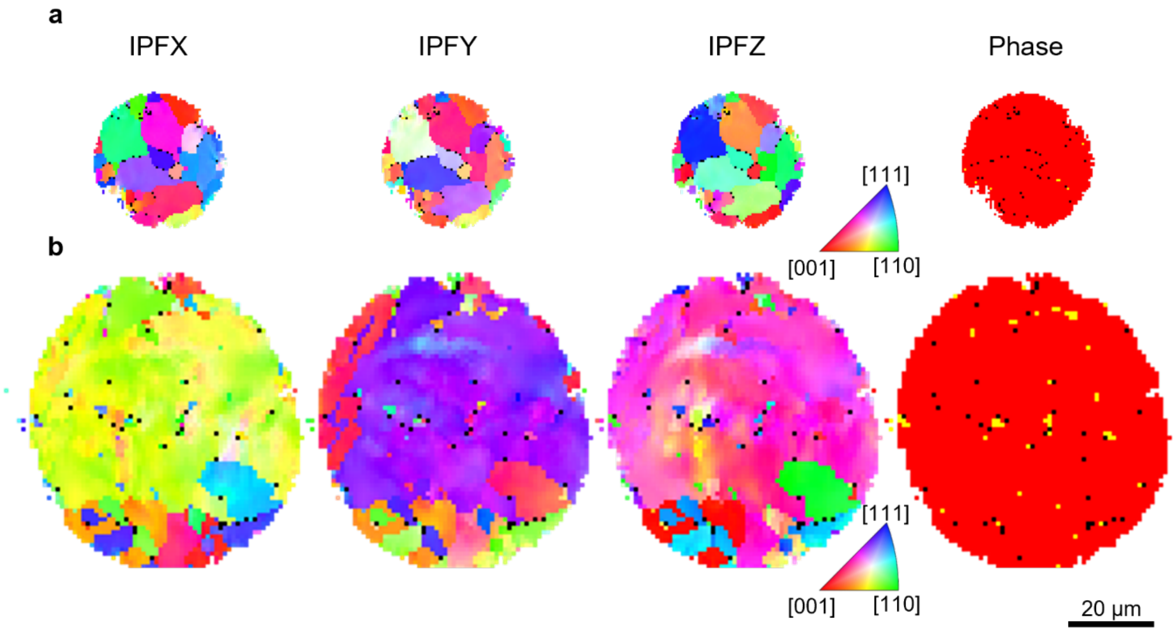

Supplementary Figure 8: Microstructure of the granulated feedstock employed for the L-DED and E-PBF systems. EBSD crystallographic orientation and phase distribution maps obtained for (a) fine-powder and (b) coarse-powder.

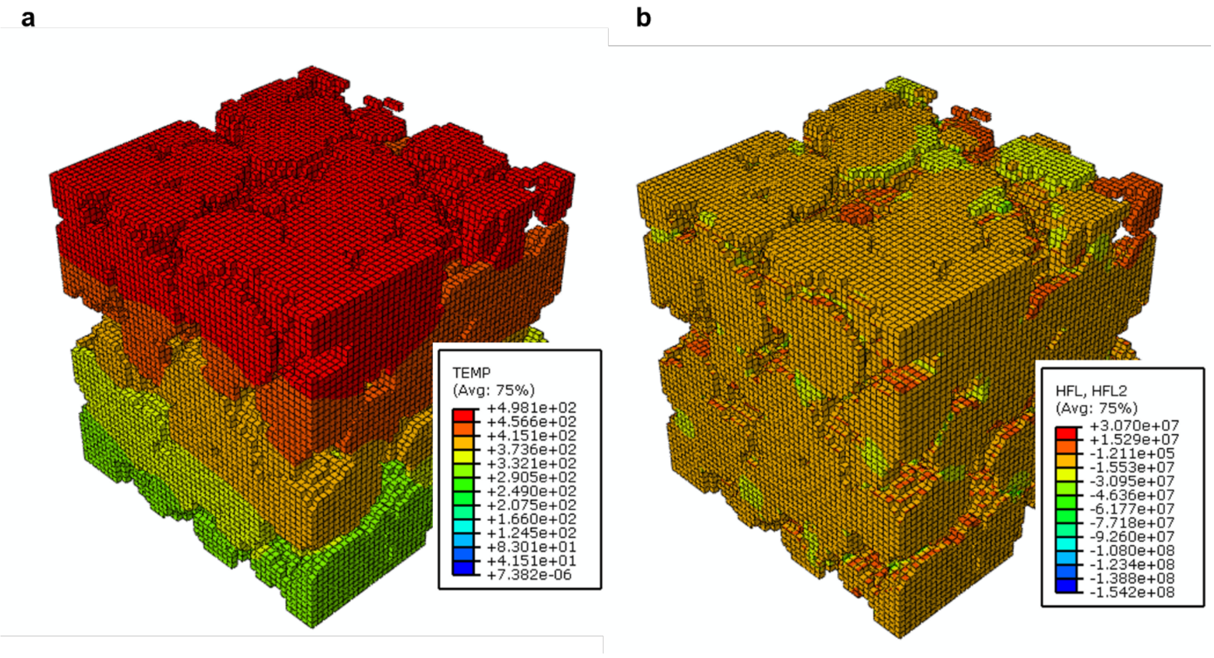

Supplementary Figure 9: Steady-state heat transfer simulations on the particulate microstructure resembling sintered powder-bed. (a) Temperature distribution contour (b) Heat flux distribution contour. The sides of the cube are 200 μm long.

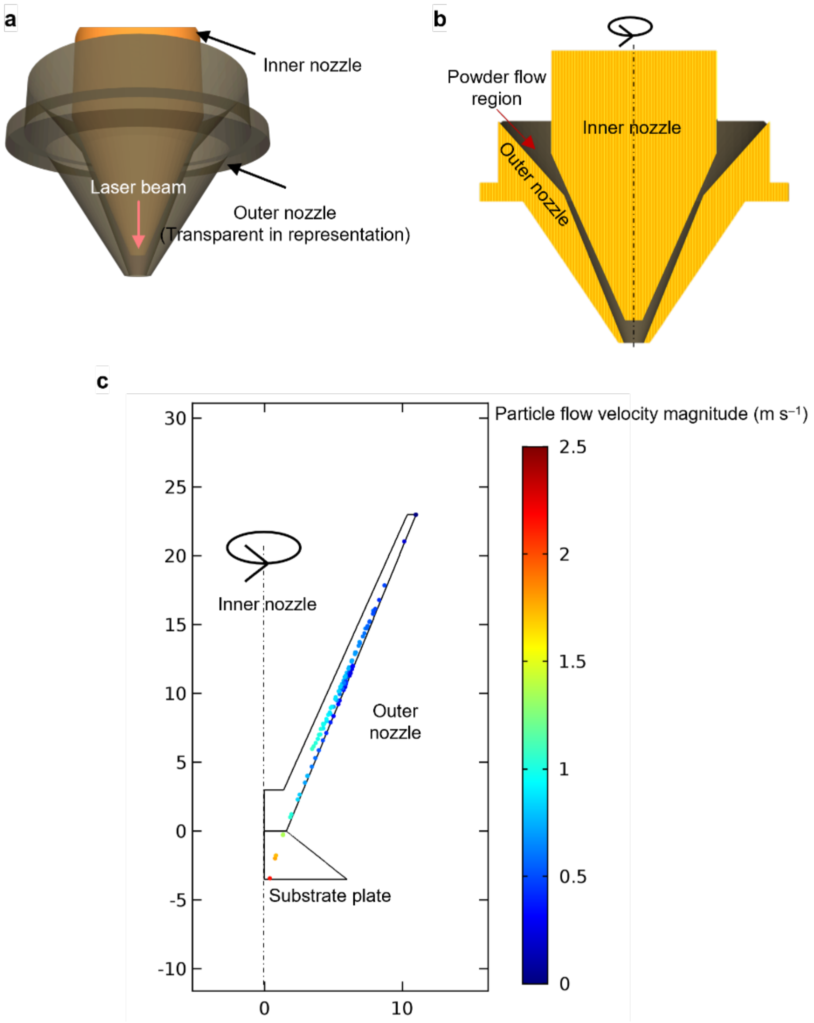

Supplementary Figure 10: DPM simulations of the fine and coarse powder flow through the nozzle assembly of the DED system employed. (a) A 3D model of nozzle assembly. (b) A sectioned face of the nozzle assembly showcasing the region of the powder flow in the BeAM Magic 800 system. (c) DPM-simulation results obtained with the contour corresponding to the particle flow velocity magnitude in m s^{-1}.

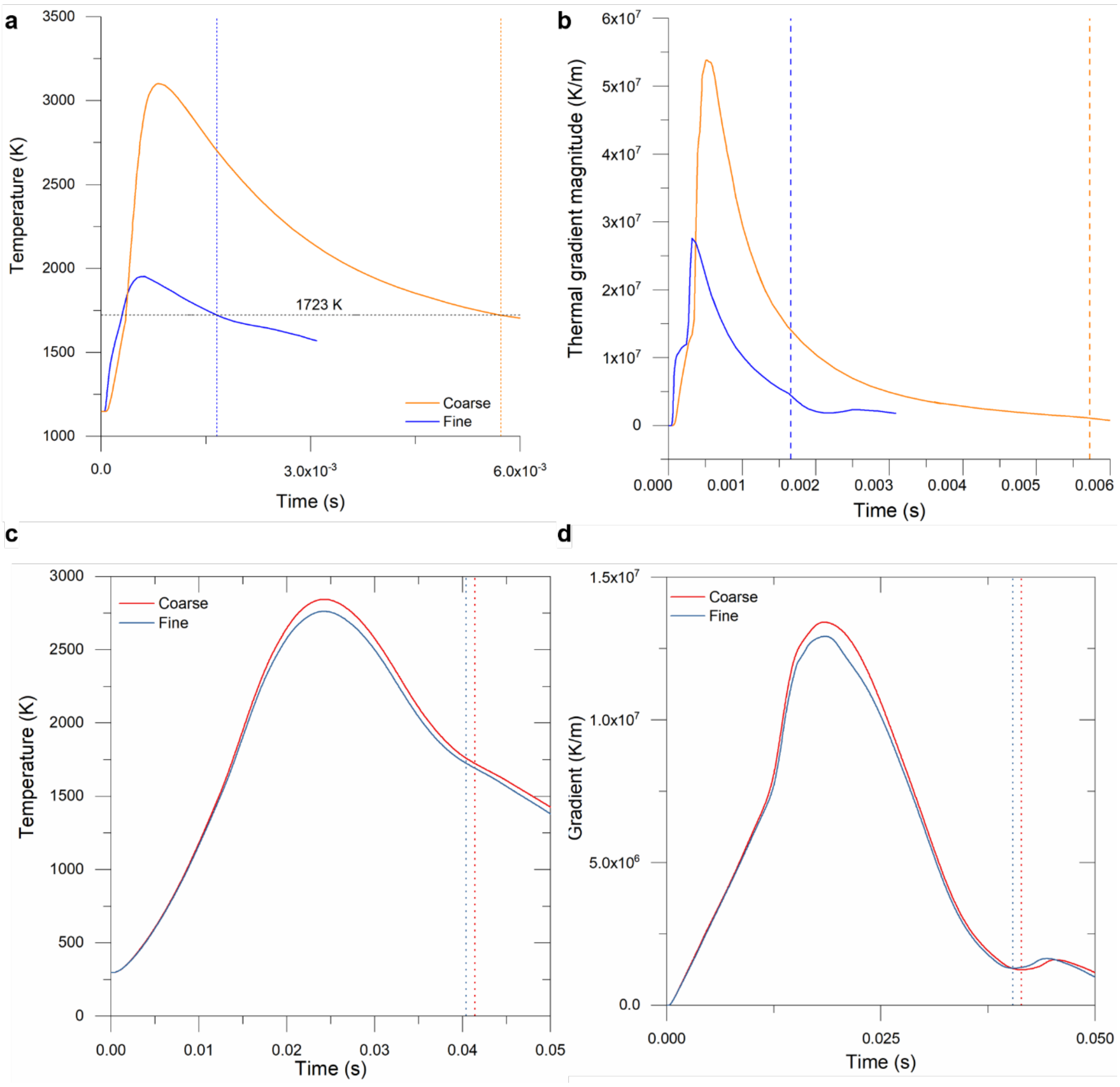

Supplementary Figure 11: Solidification parameters obtained from the FEA simulations of single tracks. (a) Temperature and (b) thermal gradient variation with time obtained from E-PBF simulations. (c) Temperature and (d) Thermal gradient variation with time obtained from L-DED simulations.

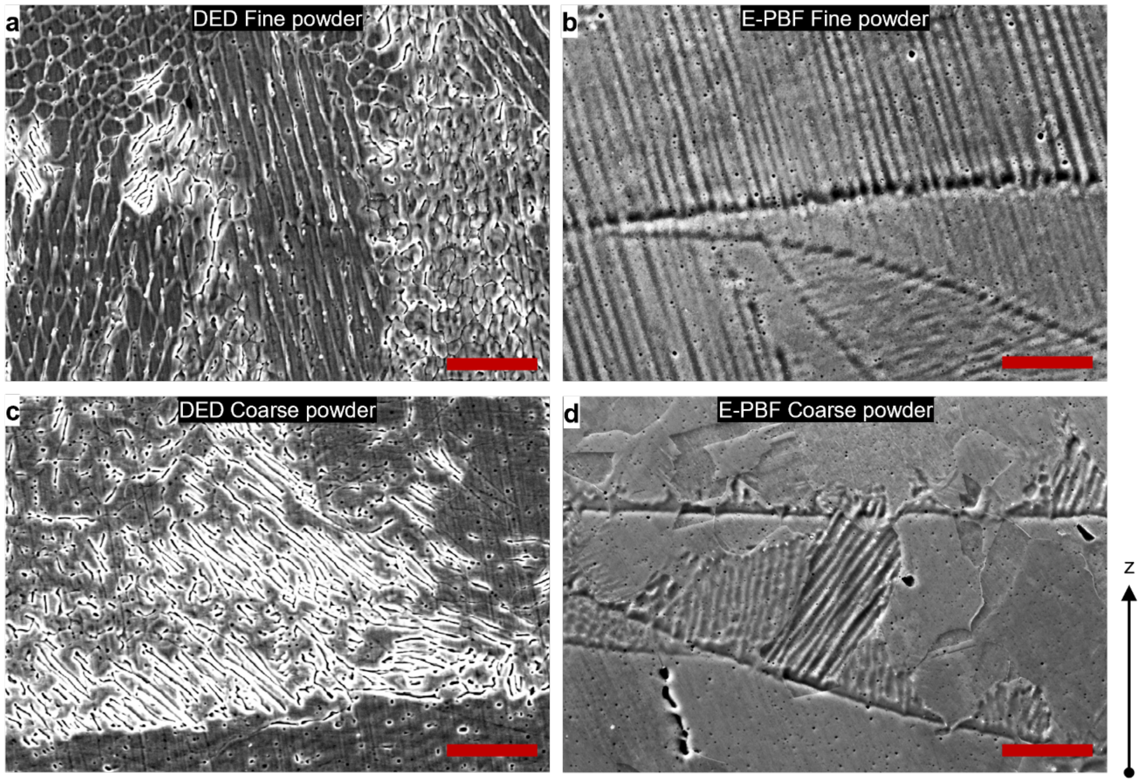

Supplementary Figure 12: Microstructural features observed in the melt pools of PF-AM processes by SEM. (a) and (b) are micrographs observed in the fine-powder samples obtained from DED and E-PBF processes, respectively. (c) and (d) are observed in the coarse-powder samples obtained from DED and E-PBF processes, respectively. Majority region of the micrograph of DED fine-powder sample is decorated with columnar dendritic sub-grain structure (a) whereas the same is on borderline of cellular-columnar dendritic in the E-PBF fine-powder samples (b). Moving towards the extreme conditions of equiaxed grain growth E-PBF coarse-powder samples have an equiaxed grains with still visible sub-grain structure (d). Farther ahead, in the DED coarse-powder samples, the sub-grain morphology begins to randomize which is evident of nearing the critical growth rate for local interfacial equilibrium assumptions [9]. The scale bars in the micrographs correspond to 10 μm in length.

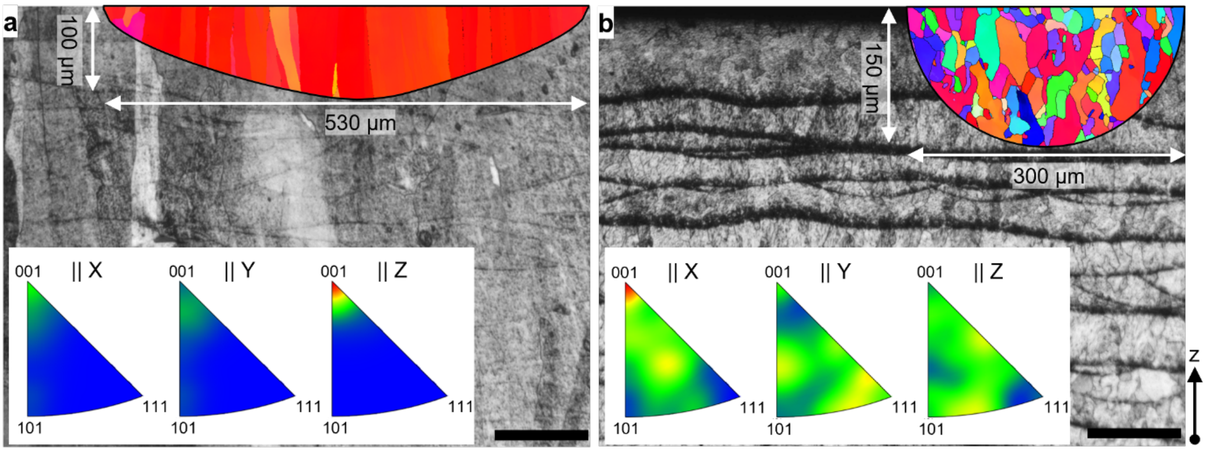

Supplementary Figure 13: Microstructure obtained by particle size-driven MPE approach in E-PBF process. (a) Fine-powder E-PBF SS316L samples show shallow melt pools with w:d of ~ 5.3 in OM image along with the representative crystallographic orientation having strong <001> crystallographic texture. (b) Coarse-powder E-PBF SS316L samples show nearly semicircular melt pool architecture favouring CET with a representative diffuse texture in one melt pool. The scale bars in the OM images are 100 µm long. The scale bars in the micrographs correspond to 100 µm in length.

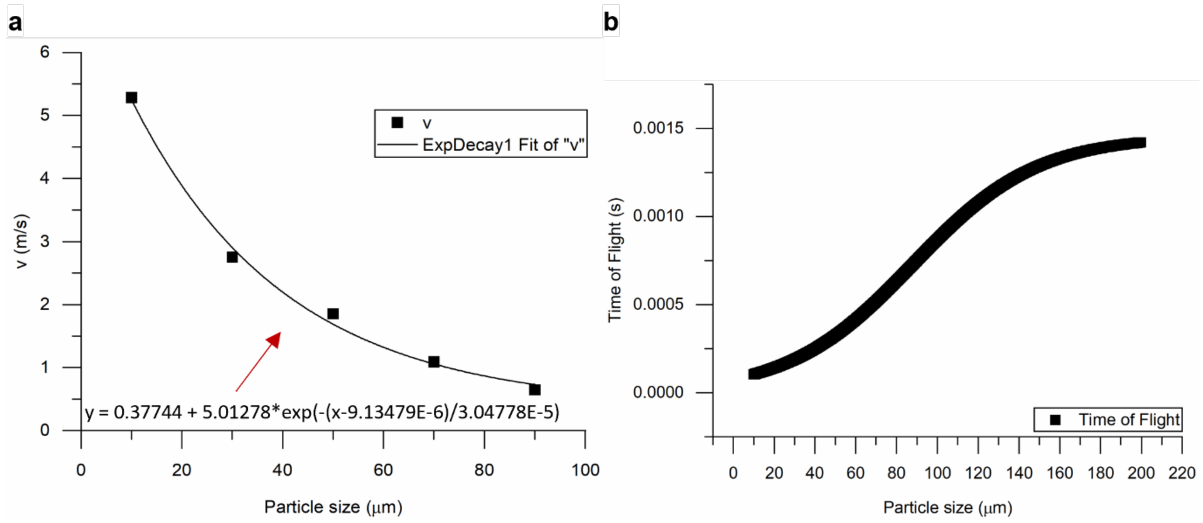

Supplementary Figure 14: Particle size dependent velocity (left) and time of flight (right) variations obtained from the DPM simulations. (a) Particle vertical speed (m s^{-1}) vs particle size (µm). Exponential decay fit equation (red arrow) employs the particle size value in m. (b) Time-of-flight variations with particle size obtained by employing the exponential decay equation shown in (a).

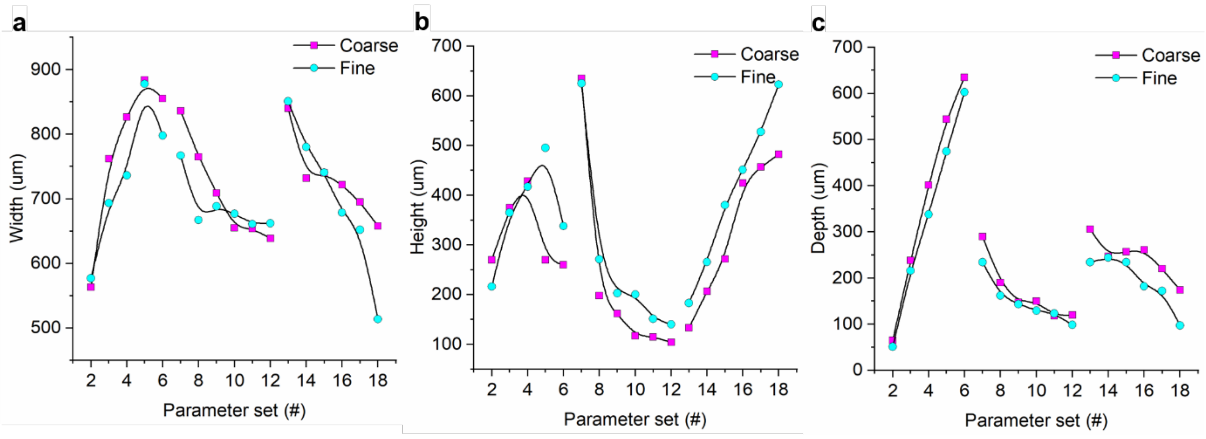

Supplementary Figure 15: Variations in the bead and melt pool dimensions with process parameters in L-DED SS316L. (a) Melt pool width, (b) Bead height, and (c) Melt pool depth variations with process parameter set. Set #2 - 6 involve increasing laser power from 200 – 600 W, #7 - 12 involve increasing scanning speeds from 500 – 3500 mm min^{-1}. #13 - 18 involve increasing powder flow rates from 1 – 6 g min^{-1}.

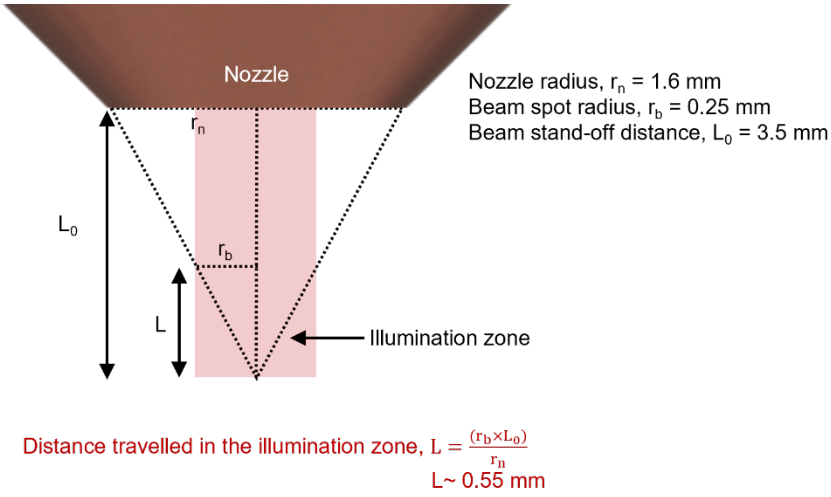

Supplementary Figure 16: Measurements of illumination zone and vertical distance travelled by the powder particles for the L-DED nozzle configuration. The time-of-flight (TOF) for the powder particles is calculated by dividing the distance travelled in the illumination zone, L by the particle speed in the vertical direction obtained by the discrete particle method (DPM) simulations.

Supplementary Tables

Supplementary Table 1 L-DED process parameter sets employed for single-track deposits for the fine- and coarse-powder feedstocks.

Parameter set	1	2	3	4	5	6	7	8	9	10	11	12	13	14	15	16	17	18
	Increasing power						Increasing scanning speed						Increasing powder flow rate					
Power (W)	100	200	300	400	500	600	300	300	300	300	300	300	300	300	300	300	300	300
Deposition speed (mm min^{-1})	1000	1000	1000	1000	1000	1000	500	1500	2000	2500	3000	3500	1000	1000	1000	1000	1000	1000
Powder flow rate (g min^{-1})	3.25	3.25	3.25	3.25	3.25	3.25	3.25	3.25	3.25	3.25	3.25	3.25	1	2	3	4	5	6

Supplementary Table 2 PSD with corresponding PBD and k obtained from the particulate microstructure simulations employed for training the ML model.

Mean (μm)	Deviation (μm)	PBD (%)	k (W $m^{-1}K^{-1}$)
81.4	31.3	53	3.85
101.0	25.6	46.5	2.63
31.8	28.2	39	1.4
116.4	11.5	49	3.61
63.6	46.0	48	0.113
98.9	41.8	40.4	1.03
57.1	35.9	48.4	2.77
95.0	12.2	52	4.97
107.0	13.9	38	1.21
38.8	9.2	40.4	1.82
66.4	24.5	48.5	2.8
33.6	24.2	37	0.57
44.5	36.4	47	2.87
20.3	9.4	37	1.89
47.9	32.5	45.3	3.42
100.5	19.6	52	4.97
84.1	6.6	46	0.0435
86.5	35.4	58	3.92
49.6	23.0	40	0.85
82.0	37.9	51	2.49

Supplementary Table 3 PSD with corresponding PBD and k obtained from the particulate microstructure simulations employed for testing the ML model. The columns with asterisk are the predicted values from the ML model.

Mean (μm)	Deviation (μm)	PBD (%)	PBD*	k (W $m^{-1}K^{-1}$)	k*
83.4	25.0	50	58.74	5.17	4.56
58.9	9.3	49	52.70	3.1	0.11
119.3	13.8	45	47.62	2.73	3.02
28.8	15.8	34	35.88	0.7	0.52
73.4	20.6	60	61.35	4.24	3.65
69.8	39.7	47	48.99	2.13	1.85

Supplementary Table 4 Calculated values of N_o parameters used for the CET curve presented in the Fig. 5 e of the main text.

	G ($K\ m^{-1}$)	n	V ($m\ s^{-1}$)	a ($K^{2.94}\ s\ m^{-1}$)	φ	N_o
Fine-EBM	4.70E+06	2.94	0.064	5.87E04	0.07	3.14E+15
Coarse-EBM	8.50E+05	2.94	0.353	5.87E04	0.69	6.64E+13
Fine-DED	1.26E+06	2.94	0.093	5.87E04	0.77	4.01E+15
Coarse-DED	1.22E+06	2.94	0.785	5.87E04	0.81	4.26E+14

Supplementary Table 5 Compositional analysis of fine and coarse powder particles using ICP-OES and combustion method (in wt.%)

	Fe	Cr	Ni	Mo	Mn	Si	Al	C	S	O	N	H
Fine powder	Bal.	15.30	10.50	1.98	1.04	0.31	0.06	0.025	0.013	0.093	0.088	0.006
Coarse powder	Bal.	16.52	10.76	2.22	0.33	0.41	0.06	0.016	0.012	0.072	0.068	0.009

Supplementary References

1. Aggarwal, A. *et al.* Role of impinging powder particles on melt pool hydrodynamics, thermal behaviour and microstructure in laser-assisted DED process: A particle-scale DEM – CFD – CA approach. *Int. J. Heat Mass Transf.* **158**, 119989 (2020).
2. Kingma, D. P. & Ba, J. Adam: A Method for Stochastic Optimization. *3rd Int. Conf. Learn. Represent. ICLR 2015 - Conf. Track Proc.* (2014).
3. Choo, H. *et al.* Effect of laser power on defect, texture, and microstructure of a laser powder bed fusion processed 316L stainless steel. *Mater. Des.* **164**, 107534 (2019).
4. Gäumann, M., Bezençon, C., Canalis, P. & Kurz, W. Single-Crystal Laser Deposition of Superalloys: Processing-Microstructure Maps. *Acta Mater.* **49**, 1051–1062 (2001).
5. Hunt, J. D. Steady state columnar and equiaxed growth of dendrites and eutectic. *Mater. Sci. Eng.* **65**, 75–83 (1984).
6. Lin, X. *et al.* Columnar to equiaxed transition during alloy solidification. *Sci. China, Ser. E Technol. Sci.* **46**, 475–489 (2003).
7. Kurz, W., Bezençon, C. & Gäumann, M. Columnar to equiaxed transition in solidification processing. *Sci. Technol. Adv. Mater.* **2**, 185–191 (2001).
8. Wu, C., Li, S., Zhang, C. & Wang, X. Microstructural evolution in 316LN austenitic stainless steel during solidification process under different cooling rates. *J. Mater. Sci.* **51**, 2529–2539 (2016).
9. Gremaud, M., Carrard, M. & Kurz, W. Banding phenomena in AlFe alloys subjected to laser surface treatment. *Acta Metall. Mater.* **39**, 1431–1443 (1991).
